# Changes in Carotid Intima-Media Thickness During the Cardiac Cycle: The Multi-Ethnic Study of Atherosclerosis

**DOI:** 10.1161/JAHA.112.001420

**Published:** 2012-08-24

**Authors:** Joseph F. Polak, Craig Johnson, Anita Harrington, Quenna Wong, Daniel H. O'Leary, Gregory Burke, N. David Yanez

**Affiliations:** 1 Department of Radiology, Tufts University School of Medicine, Boston, MA (J.F.P., A.H.); 2 Tufts Medical Center, Tufts University School of Medicine, Boston, MA (D.H.O.); 3 Collaborative Health Studies Coordinating Center, University of Washington, Seattle, WA (C.J., Q.W., N.D.Y.); 4 Division of Public Health Sciences, Wake Forest University School of Medicine, Winston-Salem, NC (G.B.)

**Keywords:** atherosclerosis, blood pressure, carotid arteries, diastole, epidemiology, risk factors, systole, ultrasonics

## Abstract

**Background:**

Common carotid artery intima-media thickness (IMT), a measure of subclinical cardiovascular disease, changes during the cardiac cycle. The magnitude of this effect and its implications have not been well studied.

**Methods and Results:**

Far-wall IMT measurements of the right common carotid artery were measured at end diastole and peak systole in 5633 individuals from the Multi-Ethnic Study of Atherosclerosis (MESA). Multivariable regression models were generated with end-diastolic IMT, peak-systolic IMT, and change in IMT during the cardiac cycle as dependent variables and traditional cardiovascular risk factors as independent variables. The average age of our population was 61.9 (45 to 84) years. Average change in carotid IMT during the cardiac cycle was 0.041 mm (95% confidence interval: 0.039 to 0.042 mm), with a mean IMT of 0.68 mm. End-diastolic IMT and peak-systolic IMT were similarly associated with risk factors. In a fully adjusted model, change in carotid IMT during the cardiac cycle was associated with ethnicity and pulse pressure (*P*=0.001) and not age, sex, or other risk factors. Chinese and Hispanics had less of a change in IMT than did non-Hispanic whites. With peak-systolic IMT reference values used as normative data, 31.3% more individuals were classified as being in the upper quartile of IMT and at high risk for cardiovascular disease than would be expected when IMT is measured at end diastole.

**Conclusions:**

Measurable differences in IMT are seen during the cardiac cycle. This affects the interpretation of IMT measurements used for cardiovascular risk assessment, given published normative data with IMT measured at peak systole.

**Clinical Trial Registration:**

URL: www.ClinicalTrials.gov. Unique identifier: NCT00063440. **(*J Am Heart Assoc*. 2012;1:e001420 doi: 10.1161/JAHA.112.001420.)**

## Introduction

The carotid artery wall intima-media thickness (IMT) is a marker of prevalent and incident cardiovascular disease.^1–2^ Carotid IMT measurements currently are done on ultrasound images of the carotid artery, preferably acquired at end diastole (ED).

A few investigations have shown that changes in IMT occur during the cardiac cycle,^3–5^ implying that arterial distension during systole is associated with a decrease in carotid IMT. The magnitude of this effect has not been documented but might have implications for cardiovascular risk assessment.

Current strategies recommending the use of carotid IMT for cardiovascular risk assessment^6^ indicate that individuals should be classified as being at high risk if their IMT is above the 75th percentile.^6–7^ However, some of the referenced IMT data have been measured on images taken at peak systole (PS).^8–9^ This implies that use of these normative data might inappropriately classify individuals as being at high risk.

We hypothesize that both peak-systolic and end-diastolic IMT (PS-IMT and ED-IMT) measurements are similarly associated with cardiovascular risk factors. We also hypothesize that use of normative data that are based on PS images (largest diameter) will overclassify individuals at risk. We investigate these issues in a community-based multiethnic cohort: the Multi-Ethnic Study of Atherosclerosis (MESA) IMT Progression Study.

## Methods

### Population

Between July 2000 and August 2002, MESA recruited and examined a multiethnic population of 6814 men and women 45 to 84 years of age with no history of clinical cardiovascular disease.^10^ MESA is a multiethnic cohort that includes non-Hispanic white, Hispanic, African American, and Chinese participants. Equal numbers of men and women were recruited from ≥2 of the 4 racial/ethnic groups at 6 field centers in the United States matched to 6 geographic regions: Baltimore City and Baltimore County, MD; Chicago, IL; Forsyth County, NC; Los Angeles County, CA; New York, NY; and St. Paul, MN. Although each center had a specific racial/ethnic recruitment goal, additional participants also were recruited from other ethnic groups to minimize confounding of ethnicity by site. Except when random-digit telephone dialing was used, an informational brochure was mailed to households within targeted regions at the 6 geographic locations. Households were contacted by telephone 14 days later to determine the language spoken in the home. A questionnaire was administered in English, Spanish, Cantonese, or Mandarin to introduce the study and to collect eligibility information. All eligible individuals are enumerated. Additional elderly members of minority groups were recruited toward the end of the recruitment period by asking already-enrolled participants to refer elderly persons to the study.

Participants were excluded if they had physician diagnosis of myocardial infarction, stroke, transient ischemic attack, heart failure, angina, or atrial fibrillation or history of any cardiovascular procedure. Weight >300 lbs, pregnancy, or any medical conditions that would prevent long-term participation were also exclusion criteria. MESA protocols and all studies described herein have been approved by the institutional review boards of all collaborating institutions. All participants gave informed consent. The analyses performed in this article are cross sectional and use the measurements made at the baseline study (July 2000 and August 2002) in 5633 of the 6814 original MESA participants who are part of the MESA IMT progression study and were seen in September 2002 through January 2004 and March 2004 through July 2005.^11^

### Study Measurements

Age, sex, race/ethnicity, and medical history were self-reported. Current smoking was defined as self-report of a cigarette in the prior 30 days. Height measurements were made in centimeters (cm), and weight was measured after an overnight fast in pounds. Resting blood pressure (BP) was measured 3 times in the seated position with a Dinamap model Pro 100 automated oscillometric sphygmomanometer (Critikon, Tampa, FL). The average of the last 2 measurements was used in analyses. The pulse pressure (systolic BP − diastolic BP) was used in the analyses. Hypertension was defined as a systolic BP ≥140 mm Hg, a diastolic BP ≥90 mm Hg, or current use of medications for BP control.

Glucose and lipids were measured after a 12-hour fast. Serum glucose was measured by rate reflectance spectrophotometry on the Vitros analyzer (Johnson & Johnson Clinical Diagnostics, Inc, Rochester, NY). The presence of diabetes mellitus was based on self-reported physician diagnosis, use of insulin or oral hypoglycemic agent, or a fasting glucose value ≥126 mg/dL.^12^ Total cholesterol was measured with a cholesterol oxidase method (Roche Diagnostics), as was high-density lipoprotein cholesterol after precipitation of non–high-density lipoprotein cholesterol with magnesium/dextran and triglycerides with Triglyceride GB reagent (Roche Diagnostics). Low-density lipoprotein cholesterol was calculated in plasma specimens that had a triglyceride value <400 mg/dL.^13^

### Carotid Artery Measurements

Participants were examined supine with the head rotated 45° toward the left side. Imaging was done in the plane perpendicular to the neck with the jugular vein lying immediately above the common carotid artery (or at 45° from the vertical if the internal jugular vein was not present). The image was centered on a 10-mm segment of the right common carotid artery at least 5 mm below (caudad to) the right common carotid artery bulb in a region free of visible plaque. A matrix array probe (M12L, General Electric, Milwaukee, WI) was used, with the frequency set at 13 MHz, with 2 focal zones, and with the frame rate set at 32 frames/second. A super-VHS videotape recording was then made for 20 seconds. Images were digitized at 30 frames/second, and automated diameter measurements were made from this video segment with the use of customized software. The diameter-versus-time curves were reviewed, and the PS (largest artery diameter) and ED (smallest artery diameter) time points were identified. These ED and PS diameter locations were then used to select 2 images, 1 at PS and 1 at ED. These selected images were used for mean far-wall common carotid artery IMT measurements via manual tracings.^14^ Interreader reproducibility was assessed by having 2 sonographers blindly select and redigitize an ED image from the 20-second videotape recording of a randomly selected set of 114 studies originally read by a third sonographer. Each sonographer then performed an IMT measurement. One sonographer reread 66 studies for an interreader correlation coefficient of 0.84, and the second sonographer reread 48 studies for an interreader correlation coefficient of 0.86.

### Statistical Analyses

The mean (and standard deviation) values of continuous variables, cardiovascular risk factors, body mass index, PS-IMT, ED-IMT, and change in IMT between diastole and systole (diastolic IMT − systolic IMT) are presented. The distribution of ED-IMT and PS-IMT values as a function of 4 age group categories (45 to 54 years, 55 to 64 years, 65 to 74 years and 75 years of age or greater) was generated. These data were also presented as a figure.

Multivariable regression models were fitted for PS-IMT, ED-IMT, and change in IMT as dependent variables with covariates age, sex, and ethnicity (minimally adjusted models). Age-, sex-, and ethnicity-adjusted regression models included the following candidate variables: smoking, diabetes, hypertension, total cholesterol and high-density lipoprotein cholesterol, systolic pressure, diastolic pressure, body mass index (kg/m^2^), and pulse pressure. Analyses that included lipid levels and BP included a variable for ongoing treatment of dyslipidemia or hypertension. Parsimonious models with age, sex, and race/ethnicity were generated with change in IMT (difference between ED-IMT and PS-IMT) as dependent variable, with the inclusion of additional variables that were associated significantly with change in IMT. This was repeated separately for men and women, forcing age and race/ethnicity into the models.

Differences between risk factor associations and PS-IMT and ED-IMT were investigated with the use of seemingly unrelated regression models.^15^

Identification of high-risk individuals was made on the basis of ED-IMT values above the 75th percentile and was compared to normative data that used the PS values. Overclassification of individuals at risk was calculated and expressed as a percentage. Results were displayed graphically by using cumulative distributions of PS-IMT and ED-IMT values.

All analyses were performed in Intercooled STATA 10.0 (StataCorp, College Station, TX). Level of statistical significance was set at *P*≤0.05. All statistical tests and associated *P* values were 2 sided.

## Results

Table 1 summarizes the prevalence of risk factors and key variables in the study's 5633 participants with complete datasets. The average age was 61.9 years, with 48% of the sample being male. The major ethnic group consisted of non-Hispanic whites (40%), followed by African Americans (26%), Hispanics (22%), and Chinese (12%). The mean carotid IMT was 0.64±0.18 mm at end systole and 0.68±0.19 mm at ED. The mean unadjusted difference between PS-IMT and ED-IMT was 0.041 mm (95% confidence interval [CI]: 0.039 to 0.042 mm).

**Table 1.** Demographics, Risk Factors, and Carotid Artery IMT in the Baseline Measurements of the MESA IMT Progression Study

Baseline Demographics, Risk Factors, and Subclinical Disease Measures	All (N=5633)
Age, n (%)	61.91 (10.14)
Sex, n (%)	
Male	2700 (48)
Female	2933 (52)
Ethnicity, n (%)	
Whites	2226 (40)
Chinese	690 (12)
Blacks	1490 (26)
Hispanics	1227 (22)
High-density lipoprotein cholesterol,* mg/dL	50.99±14.69
Low-density lipoprotein cholesterol,* mg/dL	117.22±31.06
Total cholesterol,* mg/dL	194.15±35.26
Triglycerides,* mg/dL	131.05±87.53
Lipid-lowering medications, n (%)	913 (16)
Body mass index, kg/m^2^	28.20±5.32
Height, cm	166.61±10.01
Weight, lbs	173.06±37.53
Diabetes mellitus by 2003 ADA fasting criteria algorithm, n (%)	
Normal	4182 (74)
Impaired fasting glucose	762 (14)
Untreated diabetes	146 (3)
Treated diabetes	527 (9)
SBP, mm Hg	125.88±21.16
DBP, mm Hg	71.80±10.25
Pulse pressure (SBP−DBP), mm Hg	54.08±16.91
Hypertension by JNC VI (1997) criteria, n (%)	2454 (44)
Antihypertensive medications, n (%)	2040 (36)
Cigarette smoking status, n (%)	
Never	2846 (50)
Former	2067 (37)
Current	707 (13)
Pack-years of cigarette smoking	11.10±22.22
Far-wall mean IMT, at diastole	0.68±0.19
Far-wall mean IMT, at systole	0.64±0.18
Far-wall mean IMT difference (diastole−systole)	0.04±0.05

Values are mean±SD or n (%). ADA indicates American Diabetes Association; IMT, intima-media thickness; MESA, Multi-Ethnic Study of Atherosclerosis; SBP, systolic BP; DBP, diastolic BP; and JNC VI, Sixth Report of the Joint National Committee on Prevention, Detection, and Treatment of High Blood Pressure.

* Units can be converted to SI units (mmol/L) by multiplying cholesterol values by 0.0259 and triglyceride values by 0.0113.

**Figure 1.**
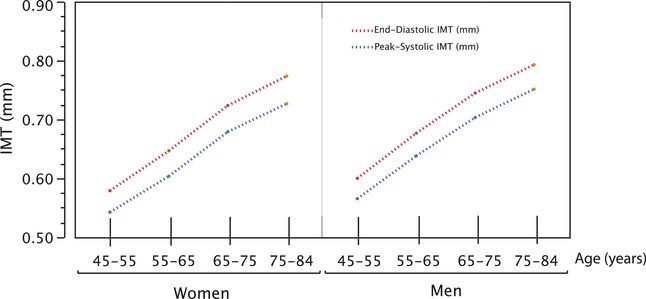
The mean common carotid artery IMT in millimeters is plotted for men and women as a function of 4 age categories. The ED-IMT values are consistently larger than the PS values. In addition, the magnitude of this difference is larger than the sex difference between men and women in the same age groups. IMT indicates intima-media thickness; ED, end diastole; and PS, peak systole.

Table 2 presents the mean PS-IMT and ED-IMT values as a function of age groups. These data also are shown graphically in Figure 1. The differences between PS-IMT and ED-IMT values are larger than the differences in IMT between men and women of the corresponding age groups.

**Table 2.** Magnitude of Differences in IMT Due to the Cardiac Cycle and Differences in IMT Between Men and Women

	Mean ED-IMT, mm, mean±SD			Diastolic–Systolic Differences, mm (95% CI)	
			
Age Group	Women	Men	IMT Difference Between Men and Women, mm (95% CI)	Women	Men
45 to 54 y	0.579±0.125	0.600±0.146	0.021 (0.008 to 0.034)	0.037 (0.034 to 0.039)	0.035 (0.032 to 0.037)
55 to 64 y	0.647±0.154	0.677±0.185	0.030 (0.013 to 0.047)	0.043 (0.044 to 0.046)	0.038 (0.035 to 0.042)
65 to 74 y	0.724±0.192	0.745±0.205	0.021 (0.002 to 0.040)	0.045 (0.042 to 0.047)	0.042 (0.038 to 0.046)
75 to 84 y	0.773±0.213	0.793±0.191	0.019 (−0.01 to 0.048)	0.047 (0.041 to 0.053)	0.042 (0.036 to 0.047)

SD indicates standard deviation; 95% CI, 95% confidence intervals for the comparisons between groups using Student *t* test; IMT, intima-media thickness; ED, end diastole.

Table 3 presents the unadjusted sex and ethnic differences in IMT between ED and PS. Men had overall smaller changes in IMT during the cardiac cycle than those of women (0.039±0.047 mm versus 0.042±0.044 mm; *P*<0.008). Differences between ethnic groups were also significant (*P*=0.005), with Chinese having the smallest changes in IMT during the cardiac cycle (0.036±0.035 mm) as compared to non-Hispanic whites (0.042±0.044 mm).

**Table 3.** Unadjusted Differences in IMT Between PS and ED for Sex and Ethnicity

95% CI for Mean	
	N	Mean	SD	Lower Bound	Upper Bound
Female	2933	0.0421	0.0440	0.0405	0.0437
Male	2700	0.0387	0.0474	0.0369	0.0405
Non-Hispanic whites	2226	0.0423	0.0436	0.0405	0.0441
Chinese	690	0.0361	0.0355	0.0335	0.0388
African American	1490	0.0409	0.0533	0.0382	0.0436
Hispanic	1227	0.0391	0.0443	0.0367	0.0416

SD indicates standard deviation. IMT indicates intima-media thickness; PS, peak systole; ED, end diastole.

Differences between male and female significant at the *P*=0.005 level; differences between ethnic groups significant at the *P*=0.011 level based on analysis of variance.

The explained variability of fully adjusted models with ED-IMT as outcome (R^2^=0.202) was similar to that for PS-IMT (R^2^=0.195). Systolic BP, age, sex, and ethnicity had small but statistically significant differences in the strength of their associations with ED-IMT and PS-IMT in age-, sex-, and ethnicity-adjusted models (Table 4).

**Table 4.** Coefficient Estimates (95% CIs) for Risk Factors in Minimally Adjusted Multivariable Regression Models

	Minimally Adjusted* Models		*P* for Differences in Predictor Coefficient From the 2 Models†
	ED-IMT	PS Velocity	
Age	0.0071 (0.0066 to 0.0075)	0.0068 (0.0063 to 0.0072)	<0.001
Ethnicity (reference non-Hispanic white)			
Chinese	−0.0092 (−0.0239 to 0.0054)	−0.0033 (−0.0176 to 0.0110)	0.020
African American	0.0544 (0.0431 to 0.0657)	0.0557 (0.0447 to 0.0668)	
Hispanic	0.0048 (−0.0071 to 0.0168)	0.0076 (−0.0041 to 0.0193)	
Sex, male	0.0254 (0.0165 to 0.0344)	0.0289 (0.0201 to 0.0377)	0.004
Body mass index	0.0027 (0.0018 to 0.0036)	0.0027 (0.0018 to 0.0036)	0.833
High-density lipoprotein cholesterol,‡ mg/dL	−0.0009 (−0.0012 to −0.0006)	−0.0009 (−0.0013 to −0.0006)	0.616
Total cholesterol (adjusted for lipid-lowering medication use), mg/dL	0.0003 (0.0001 to 0.0004)	0.0003 (0.0001 to 0.0004)	0.639
Diabetes mellitus (reference: normal)			
Impaired fasting glucose	0.0133 (−0.0002 to 0.0267)	0.0132 (0.0000 to 0.0263)	0.140
Untreated diabetes	0.0361 (0.0078 to 0.0645)	0.0303 (0.0025 to 0.0581)	
Treated diabetes	0.0357 (0.0198 to 0.0515)	0.0317 (0.0161 to 0.0472)	
Cigarette smoking status			
Former	0.0062 (−0.0050 to 0.0174)	0.0069 (−0.0041 to 0.0178)	0.606
Current	0.0187 (0.0027 to 0.0347)	0.0173 (0.0016 to 0.0329)	
Adjusted for pack-years	0.0002 (−0.0001 to 0.0004)	0.0002 (0.0000 to 0.0005)	
Hypertension	0.0341 (0.0244 to 0.0437)	0.0314 (0.0219 to 0.0408)	0.038
SBP (adjusted for antihypertension medication), mm Hg	0.0014 (0.0012 to 0.0017)	0.0013 (0.0011 to 0.0015)	<0.001
DBP (adjusted for antihypertension medication), mm Hg	0.0009 (0.0004 to 0.0014)	0.0008 (0.0004 to 0.0013)	0.287
Pulse pressure (adjusted for antihypertension medication), mm Hg	0.0022 (0.0019 to 0.0025)	0.0020 (0.0017 to 0.0023)	<0.001

IMT indicates intima-media thickness; ED, end diastole; MESA, Multi-Ethnic Study of Atherosclerosis; SBP, systolic BP; and DBP, diastolic BP.

* Minimally adjusted models are adjusted for age, ethnicity, and sex only.

† *P* value reflects seemingly unrelated regression analysis test result of coefficient predicting ED-IMT = coefficient predicting PS-IMT.

‡ Convert coefficient to SI units (mmol/L) by multiplying cholesterol values by 0.0259.

Multivariable models with difference in IMT during the cardiac cycle as the outcome showed that ethnicity and pulse pressure were the only risk factors associated with difference in IMT between systole and diastole (Table 5). A 10–mm Hg increase in pulse pressure corresponded to a 0.0022-mm (95% CI: 0.0013 to 0.0032 mm) decrease in IMT during the cardiac cycle.

**Table 5.** Coefficient Estimates (95% CIs) for Risk Factors Predicting the Difference Between IMT at ED and IMT at PS as the Outcome Variable in Multivariable Regression Models

	Minimally Adjusted* Model	Fully Adjusted Model†
Age	0.0003 (0.0002 to 0.0004)§§	0.0001 (0.0000 to 0.0003)
Ethnicity (reference non-Hispanic white)		
Chinese	−0.0059 (−0.0091 to −0.0027)§§	−0.0069 (−0.0105 to −0.0033)§§
African American	−0.0013 (−0.0046 to 0.0019)	−0.0032 (−0.0067 to 0.0002)
Hispanic	−0.0028 (−0.0059 to 0.0003)	−0.0039 (−0.0072 to −0.0006)#
Sex, male	−0.0035 (−0.0059 to −0.0011)¶	−0.0017 (−0.0044 to 0.0011)
Body mass index	0.0000 (−0.0002 to 0.0003)	…
High-density lipoprotein cholesterol‡, mg/dL	0.0000 (−0.0001 to 0.0001)	…
Total cholesterol (adjusted for lipid-lowering medication use), mg/dL	0.0000 (0.0000 to 0.0000)	
Diabetes mellitus by 2003 ADA fasting criteria algorithm (reference Normal)		
Impaired fasting glucose	0.0001 (−0.0039 to 0.0041)	
Untreated diabetes	0.0058 (−0.0052 to 0.0168)	
Treated diabetes	0.0040 (−0.0004 to 0.0084)	
Cigarette smoking status (adjusted for pack-years)		
Reference: never	−0.0006 (−0.0035 to 0.0022)	…
Former	0.0014 (−0.0029 to 0.0057)	…
Current	0.0000 (−0.0001 to 0.0000)	…
Hypertension by JNC VI (1997)	0.0027 (0.0001 to 0.0054)#	…
SBP (adjusted for antihypertension medications), mm Hg	0.0001 (0.0001 to 0.0002)§§	…
DBP (adjusted for antihypertension medications), mm Hg	0.0001 (−0.0001 to 0.0002)	…
Pulse pressure (adjusted for antihypertension medications), mm Hg	0.0002 (0.0001 to 0.0003)§§	0.0002 (0.0001 to 0.0003)§§

IMT indicates intima-media thickness; ED, end diastole; MESA, Multi-Ethnic Study of Atherosclerosis; SBP, systolic BP; and DBP, diastolic BP.

* Minimally adjusted models are adjusted for age, ethnicity, and sex only.

† Pulse pressure significantly associated with cardiac cycle differences in IMT. Ethnicity (Chinese and Hispanic are different than non-Hispanic whites) remains a statistically significant predictor in the model.

‡ Convert coefficient to SI units (mmol/L) by multiplying cholesterol values by 0.0259.

§§ Significant at the 0.001 level.

# Significant at the 0.01 level.

¶ Significant at the 0.05 level.

We performed additional analyses stratifying for sex. For men as well as for women, pulse pressure remained the only risk factor associated with change in IMT during the cardiac cycle. The association with race/ethnicity was no longer significant. For women, a 10–mm Hg pulse pressure increase corresponded to a 0.0022-mm decrease in IMT (95% CI: 0.0011 to 0.0034 mm). The association with race/ethnicity was no longer significant. For men, a 10–mm Hg pulse pressure increase corresponded to a 0.0024-mm decrease in IMT (95% CI: 0.0008 to 0.0039 mm).

**Figure 2.**
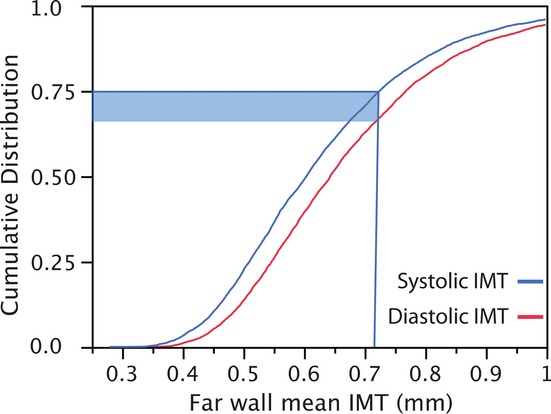
The cumulative distribution of common carotid artery IMT values based on the PS values as done in the Atherosclerosis Risk in Communities (ARIC) study is shown as the curve to the left. When an IMT measurement is made at ED, the 75th percentile cutpoint for IMT values is shifted down, increasing the number of individuals classified as being at high risk (blue area). Individuals in the shaded area are inappropriately classified as being in a high-risk category. In this case, 31.3% of individuals in the third quartile are classified as being in the upper quartile, equivalent to a relative increase of 31.3% in the high-risk group defined as having an IMT value >75% cutpoint. IMT indicates intima-media thickness; PS, peak systole; and ED, end diastole.

The IMT interquartile ranges for ED images (0.316 to 0.547 mm, 0.547 to 0.644 mm, 0.744 to 0.769 mm, and ≥0.769 mm) were consistently larger than for PS-IMT values (0.279 to 0.510 mm, 0.510 to 0.604 mm, 0.604 to 0.0725 mm, and ≥0.725 mm). There were 1848 individuals with ED-IMT values above the 75th percentile for systolic IMT, as compared to an expected 1408 individuals (Figure 2), for an overestimation of 31.3% (440/1408 individuals).

## Discussion

We have found that the difference in common carotid artery IMT between ED and PS is on average 0.041 mm. The principal predictors of this difference are pulse pressure and ethnicity. We also have found that the associations of risk factors with ED-IMT and PS-IMT are similar. We also note that adoption of IMT measurements made at ED when compared to normative data based on PS-IMT might lead to an overestimation of 31.3% of individuals at high risk for cardiovascular disease.

Before this study, there had not been a systematic evaluation of how much the phase of the cardiac cycle changes the IMT measurement. In vitro studies had suggested that the artery wall was noncompressible.^16–17^ This implied, on the basis of conservation of mass and lack of longitudinal stretching, that the wall thickness and therefore IMT would decrease as the artery diameter increased during systole. Selzer et al^4^ showed that common carotid IMT was lower at PS than for ED by an average of 5.3% in 24 individuals. Devereux et al^18^ reported a 5.3% decrease in carotid IMT with high-resolution M-mode imaging. However, Baldassarre et al^19^ studied a small sample of 14 subjects and did not detect any systematic difference in IMT during the cardiac cycle.

We estimate the change in IMT during the cardiac cycle to be on average 0.041 mm. We also show for the first time that the difference between ED-IMT and PS-IMT is associated with pulse pressure and ethnicity but not with age, sex, or any other traditional cardiovascular risk factor. On the basis of these observations, the distending pressure on the artery wall is the principal contributor to the difference in IMT between systole and diastole. Ethnic differences are described for the first time and could represent inherent differences in the structure of the arterial wall.

Previous IMT studies have (1) underestimated the role of the cardiac cycle^20^, (2) used the R wave to gate the IMT measurement to ED^21^, or (3) used a visual evaluation of the smallest diameter to identify the ED frame.^22^ The carotid image used for IMT measurements often is selected after review of a cine buffer of images stored in the ultrasound device or after review of videotaped images. The image frame closest to ED typically is selected by review of the images and by picking the image in which the diameter of the artery appears the smallest. Our findings show marginal differences in the associations of risk factors with ED-IMT and PS-IMT measurements. The difference in the explained variability of the models is small, at 0.74%, and is unlikely to affect the associations between risk factors and IMT within a given study. However, if results of different IMT studies are to be combined, appropriate adjustments might be needed.

Strengths of our study include the multiethnic composition of the cohort, the use of a highly standardized IMT imaging protocol, and the use of a high-resolution ultrasound imaging device. In addition, the data were acquired from the far wall of the common carotid artery, a preferred site when consideration is being given to the use of IMT for cardiovascular risk assessment.^8^ Weaknesses include the possibility that associations between change in IMT and risk factors might have been underestimated because of the small magnitude of the change being assessed and the variability of the measurement process. We also are limited by the fact that only the right carotid artery was studied. However, IMT evaluations restricted to the right carotid are reproducible, have predictive power for outcomes, and also are useful for assessing the response to lipid-lowering therapies.^21,23^ We have shown that cardiovascular risk factors are associated with our IMT measurements, such that 20% of the variability in IMT can be accounted for. This is within the expected range reported in prior publications that included the right and left common carotid arteries. In these studies, cardiovascular risk factors accounted for up to 27% of IMT variability in middle-age individuals^24^ and ≍17% for individuals ≥65 years of age.^25^ This issue requires further clarification because derived IMT normative data are different for the right and left common carotid arteries.^8^ It is not clear whether the IMT values used for risk assessment should be derived from either the right or left common carotid arteries or whether an average of both values should be considered.

Asymptomatic individuals can be classified as being at high risk for cardiovascular disease if their IMT is above the 75th percentile.^6–7^ A panel of the American Heart Association and American College of Cardiology Foundation on cardiovascular risk in asymptomatic individuals gave a Class IIa recommendation for the use of carotid IMT for cardiovascular risk assessment^6^ and referenced a source of normative data.^8–9^ However, these source IMT data were measured on PS images.^9^ According to our data, use of these IMT normative data might classify patients inappropriately as being at high risk and increase their likelihood of undergoing further investigation or treatment when their IMT is measured at ED. Although it is clear that Atherosclerosis Risk in Communities (ARIC) IMT values acquired at PS^26^ have predictive power for cardiovascular events,^27^ the same is true for IMT measurements made at ED.^28^

We recognize that the magnitude of this effect is small given the inherent variability of IMT measurements, but the effect is a quantifiable one nonetheless. In addition, the differences in IMT between PS and ED are greater than those seen between men and women (Table 2, Figure 1). Because men and women have different sets of normative data, it would seem logical that IMT normative values should also include a correction for the phase of the cardiac cycle.

We conclude that IMT is slightly larger at ED than it is at PS in part because of pulse pressure. Our observations confirm the need to control for the phase of the cardiac cycle when change in IMT is measured over time. The magnitude of the difference in IMT during the cardiac cycle can affect risk stratification in asymptomatic individuals because of mismatch between acquisition protocol and IMT normative data.
